# Adenocarcinoma of Mullerian Origin Presenting as Small Bowel Obstruction

**DOI:** 10.7759/cureus.32645

**Published:** 2022-12-17

**Authors:** Reema Alrasheed, Alanoud Alharkan, Bassam Alhassan, Assem Alrumeh, Bandar Ali

**Affiliations:** 1 Department of General Surgery, Prince Sultan Military Medical City, Riyadh, SAU; 2 College of Medicine, Princess Nourah Bint Abdulrahman University, Riyadh, SAU; 3 Department of Pathology, Prince Sultan Military Medical City, Riyadh, SAU

**Keywords:** rare tumor, extra-uterine müllerian tumors, small-bowel obstruction, jejunal adenocarcinoma, mullerian tumor

## Abstract

We report a case of an 84-year-old female with a history of total abdominal hysterectomy with bilateral salpingo-oophorectomy 20 years previously who presented with small bowel obstruction. Computed tomography (CT) scan with contrast showed a large mass in the mesentery of the small bowel. Exploratory laparotomy was performed, and segmental resection of 25 cm of small bowel with the mesenteric mass was performed. The histopathological features were suggestive of recurrent adenocarcinoma of Mullerian origin. The patient was offered palliative chemotherapy and referred to oncology and palliative care as a part of multidisciplinary treatment.

## Introduction

Small bowel obstruction (SBO) is a surgical emergency associated with many pathological processes. Adhesions are the most common cause in patients with surgical histories. Other common causes of SBO include hernias, volvulus, and tumors [1]. There are multiple types of small bowel tumors implicated in the condition including gastrointestinal stromal tumors (GIST), lymphomas, and small bowel adenocarcinomas. The most frequently affected part of the gastrointestinal system is the duodenum, which accounts for between 55% and 82% of all cases. The jejunum and ileum are also frequently affected by the condition with 11-25% and 7-17% prevalence respectively [2]. Symptoms of small bowel adenocarcinoma include abdominal discomfort, nausea, pain, vomiting, and weight loss. The patients are also likely to develop intestinal obstruction and gastrointestinal bleeding [2]. However, these symptoms are vague, and an accurate diagnosis of the condition based on symptoms alone could be challenging [3]. Moreover, small bowel obstruction could be associated with adenocarcinoma of Mullerian origin, a fatal rare form of the disease [3]. The condition resembles the papillary serous adenocarcinoma of the ovary and was traditionally referred to as the mesothelioma of the pelvic peritoneum. It is commonly seen among postmenopausal females aged between 56 and 62 years old and is a representation of peritoneal carcinomatosis that has no known primary site of origin [3]. We report a case of an 84-year-old woman suffering from small bowel obstruction caused by adenocarcinoma of Mullerian origin.

## Case presentation

**Figure 1:**
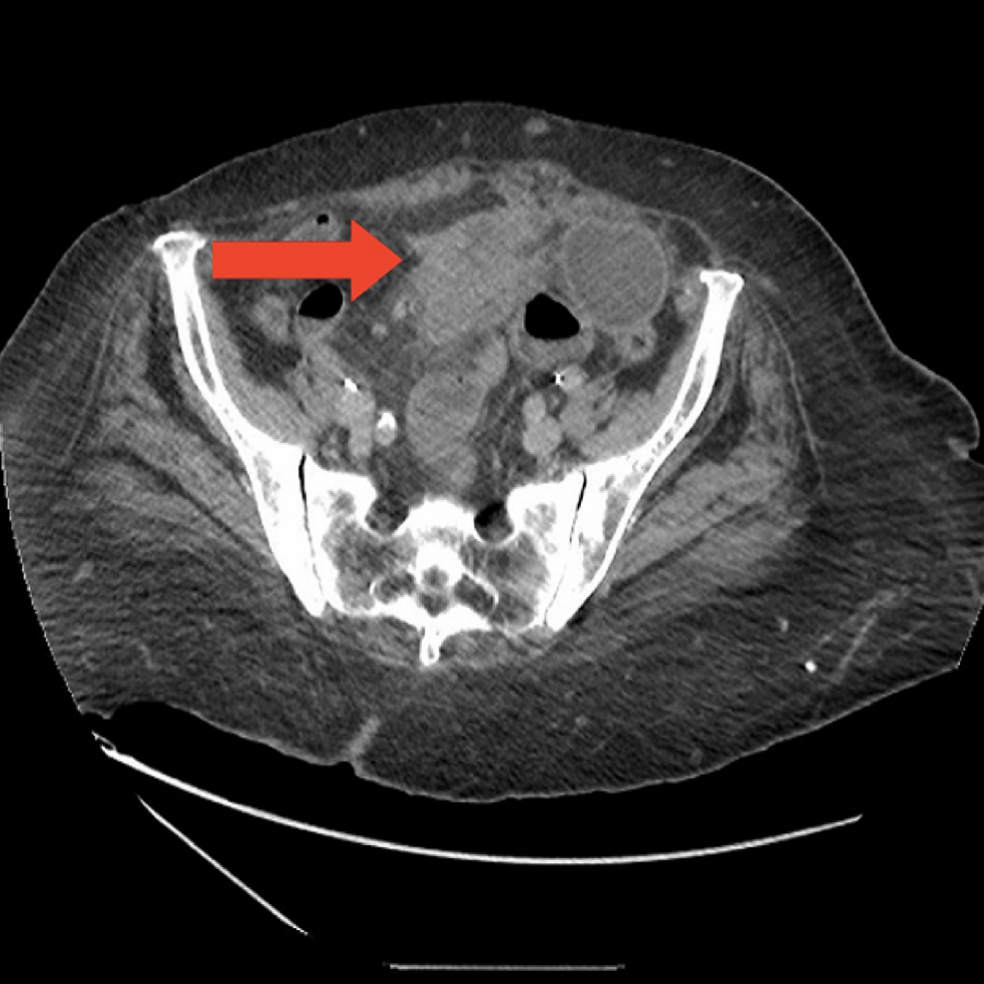
CT Axial view showing the mass (red arrow)

**Figure 2:**
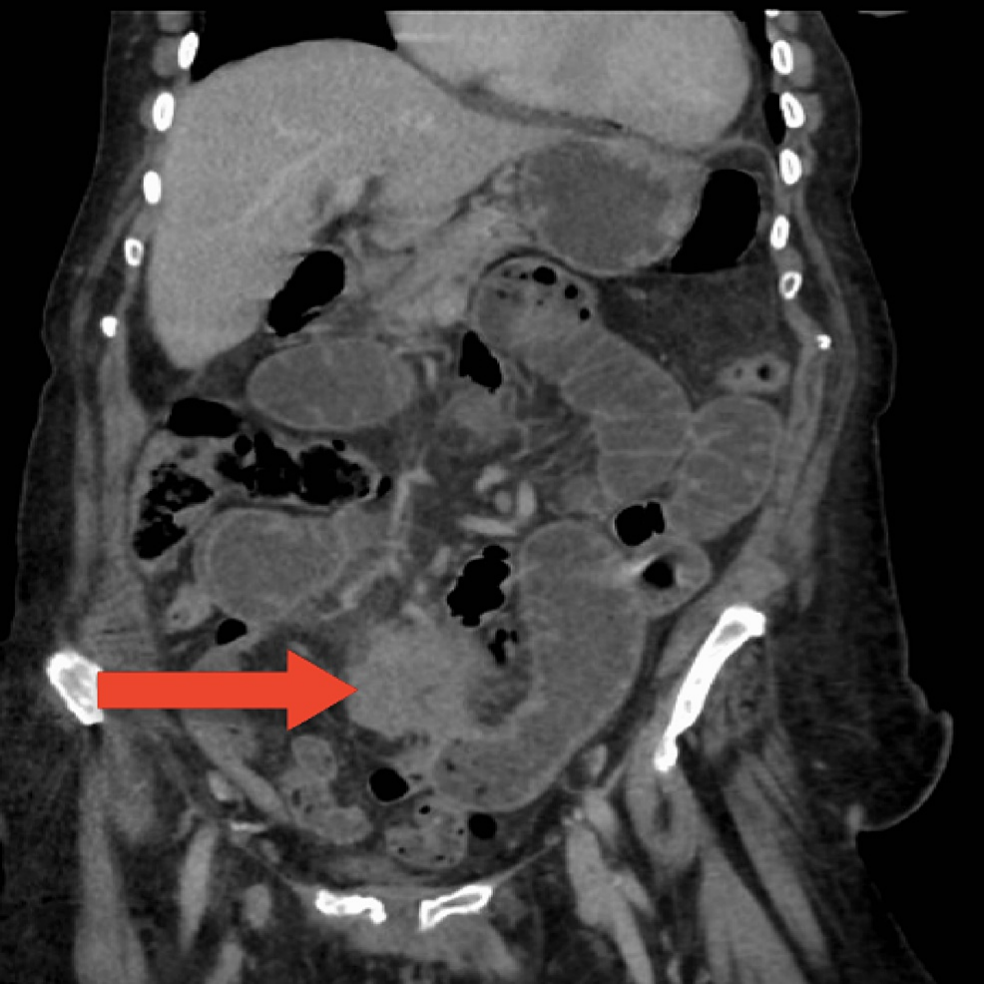
CT coronal view showing the mass (red arrow)

**Figure 3:**
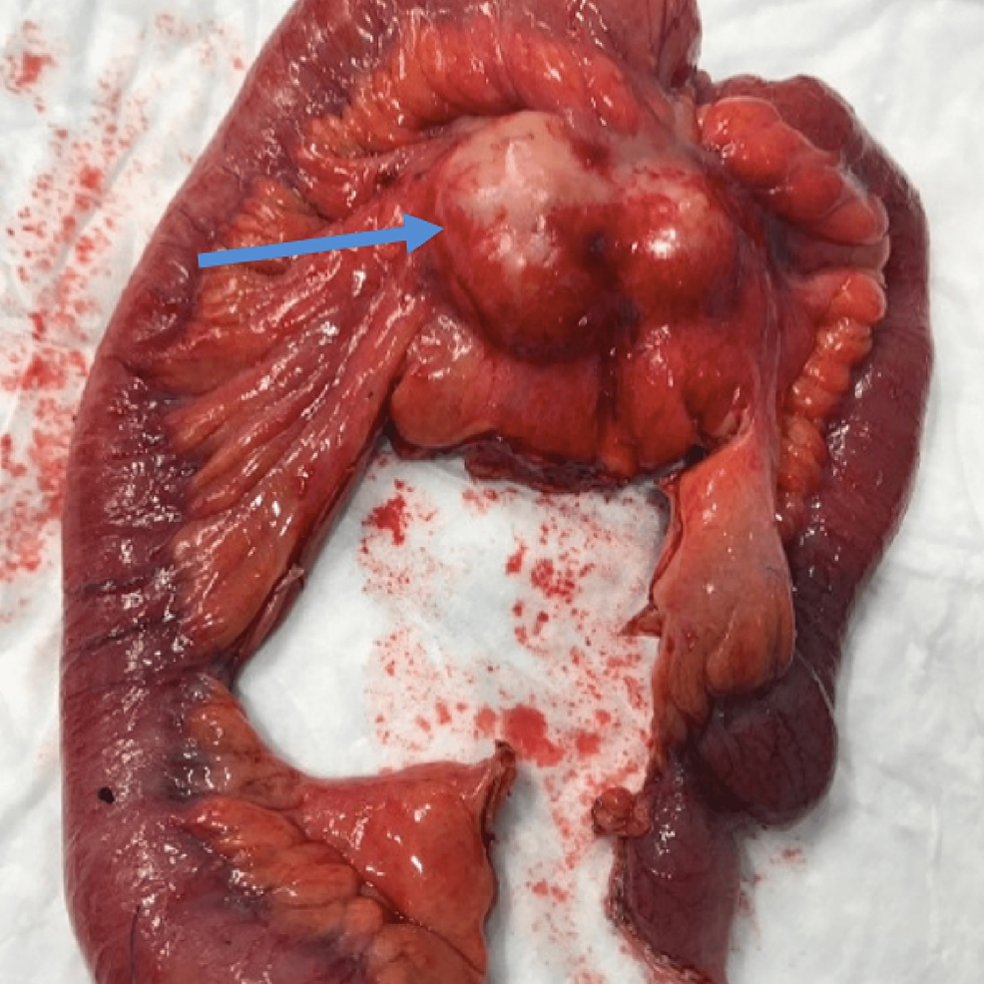
The resected bowel with its mesentery showing the mass (blue arrow)

An 84-year-old comorbid female presented to the emergency department with a one-week history of progressive and colicky epigastric pain for one week. The pain was not associated with any specific relieving or aggravating factors and was associated with a two-day history of nausea, and non-bilious vomiting. There was no history of bowel motion changes, melena, fever, night sweat, loss of weight, or change in appetite. As per her past medical history, she is known to have arterial hypertension and hypothyroidism which were both controlled with medications. Regarding surgical history, she underwent a total abdominal hysterectomy with bilateral salpingo-oophorectomy (TAH+BSO) more than 20 years ago for exophytic adenocarcinoma which was poorly differentiated, with negative lymph nodes. There was no significant family history or social history. On examination, the patient was hemodynamically stable and was afebrile. The abdomen appeared mildly distended and soft but with mild tenderness mainly in the mid-abdomen, with normal bowel sounds. No masses were palpable on examination. The patient refused digital rectal examination. Initial laboratory investigations were unremarkable. Computed tomography (CT) of her abdomen and pelvis with oral and intravenous contrast revealed a large outer-lobulated, heterogeneously enhancing, mesenteric soft tissue mass lesion at midline and left para midline space of the infra-umbilical region with a size of 6x4x3 cm, with a mass effect in term of proximal high-grade small bowel loops obstruction (Figures 1-2). The decision was taken to admit the patient, keeping her nothing by mouth, with good hydration, and a nasogastric tube (NGT) was inserted. She then underwent exploratory laparotomy. A midline incision was made, and the abdomen was explored, upon exploring a large mass measuring 6x6 cm was found in the mesentery of the small bowel. The mass was firm and fixed to the wall and the mesentery of the mid-jejunum with peritoneal deposits. Segmental resection of 25 cm of small bowel with the mesenteric mass was done (Figure 3).

**Figure 4:**
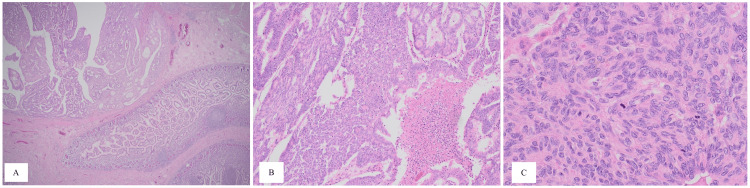
Histopathological examination of the specimen at low-power view (A) shows a well-defined neoplastic tumor with adjacent normal small bowel mucosa. At intermediate-power view (B) the neoplastic cells forming glands are shown arranged in cribriform, microacinar, and papillary patterns. At high-power view (C) marked nuclear pleomorphism and high mitotic rate can be seen.

**Figure 5:**
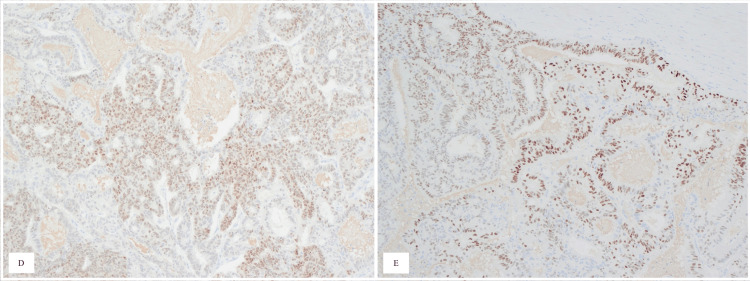
Immunohistochemical examination of the specimen shows focal nuclear staining for PAX8 (D), and ER (E).

A side-to-side anastomosis was performed, and the specimen was sent to the pathology department. Postoperative recovery was uneventful; the patient was started on a diet, gradually from liquid to solid food as tolerated, then she was discharged from the hospital on Day 6 in good health with close outpatient follow-up. In her visit to the clinic two weeks after discharge, the patient was tolerating feeding well, passing normal bowel motion and the wound was clean and healed. However, the pathologist's report showed that the resected specimen demonstrated high-grade adenocarcinoma with tumor-free resection margins. The tumor cells were positive for CK7, PAX8 (focal), ER (focal), and vimentin (focal); other immunostains including CK20, CDX2, WT1, P16, and synaptophysin were negative. The histopathological and immunohistochemical examination was consistent with recurrent/metastatic adenocarcinoma of Mullerian origin (Figures 4-5). The patient was offered palliative chemotherapy and referred to oncology and palliative care as a part of multidisciplinary treatment.

## Discussion

Adenocarcinoma of Mullerian origin is uncommon and is likely to present in post-menopausal females in their fifth and sixth decades of life [4]. Small bowel adenocarcinomas are asymptomatic in their early stages [1]. However, in the late stage, they usually present with vague clinical symptoms such as abdominal pain and ascites [4]. On the other hand, in our case, the patient presented with epigastric pain and bowel obstruction clinically and radiologically.

There are multiple theories in regard to the development of adenocarcinoma of Mullerian origin. Some include genetic alteration including BRCA 1 or p53 mutations [5,6]. The genetic screening was not done for our patient. In our case, the presentation is unique as the patient already underwent TAH+BSO 20 years ago, which revealed poorly differentiated adenocarcinoma. The mass itself was exhibiting a mass effect over the small bowel loop causing an SBO. This diagnosis of adenocarcinoma of Mullerian origin is made by histological examination, which shows an ovarian serous tumor. Moreover, immunohistochemistry helps in differentiating it from primary ovarian serous neoplasm with a positive CK20, especially if the immune stain was also negative for CK7, WT1, and PAX8; however, it is not confirmatory [3,6]. However, in our case immunohistochemistry was positive for PAX8, CK7, and ER. Interestingly it was also negative for CK20 and WT1 in comparison to the previous literature [3,6]. Moreover, adenocarcinoma of Mullerian origin can be diagnosed after radiological evidence of unremarkable gynecological organs, which in our case was not possible because of the previous surgical history of total abdominal hysterectomy with bilateral salpingo-oophorectomy [7]. The prognosis unfortunately is bad and worsens with the advancing stages especially stage III/IV [8]. It can be managed not only by cytoreductive surgery but also with chemotherapy such as cisplatin [8].

## Conclusions

Small bowel obstruction is one of the most common surgical emergencies, although rare causes can be encountered as well. In our case, metastatic adenocarcinoma of Mullerian origin after resecting the primary tumor more than 20 years back was the cause of the obstruction. Following final histopathological reports are a crucial part of the management. Also, a multidisciplinary team should be involved, which gives the patients the best medical care available.
